# Weighing the Cost and Benefit of Transcranial Direct Current Stimulation on Different Reading Subskills

**DOI:** 10.3389/fnins.2016.00262

**Published:** 2016-06-07

**Authors:** Jessica W. Younger, Melissa Randazzo Wagner, James R. Booth

**Affiliations:** ^1^Department of Communication Sciences and Disorders, Northwestern UniversityEvanston, IL, USA; ^2^Department of Communication Sciences and Disorders, University of Texas at AustinAustin, TX, USA; ^3^Department of Communication Sciences and Disorders, Teachers College, Columbia UniversityNew York, NY, USA

**Keywords:** reading intervention, transcranial direct current stimulation, parietal lobes, sight word efficiency, rhyming

## Abstract

Adults struggling with low reading skills are underserved by limited available treatments. While brain stimulation techniques such as transcranial direct current stimulation (tDCS) has the potential to improve a variety of cognitive functions, little work has been done examining its potential to treat reading disabilities. Research on the effects of tDCS on reading abilities has been somewhat inconsistent perhaps in part due to discrepancies between studies in the nature of the tasks. In the current study, we examined the effect of tDCS to the left inferior parietal lobe (L IPL) on two reading tasks in low-to-average readers. We compared performance on a sight word efficiency (SWE) task and a rhyme judgment task before and after either stimulation to the L IPL, right superior parietal lobe (R SPL), or sham stimulation. Readers who received stimulation to the L IPL showed greater improvements on the SWE task, but less improvement on the rhyme judgment task compared to the R SPL and sham groups. This study demonstrates for the first time both a positive and negative effect of stimulation under the same stimulation parameters within the same participants. The results highlight the need to consider multiple tasks when assessing the potential of using tDCS as a treatment.

## Introduction

Over the last decade, interest in using brain stimulation techniques as a therapeutic tool to treat cognitive impairment in adults has received increasing attention (Dubljević et al., 2014). Brain stimulation is a non-invasive method using electrical currents to alter the firing potential of neurons in the affected area. While there are a variety of techniques that can be used to stimulate the brain, two primary techniques are transcranial magnetic stimulation (TMS) and transcranial direct current stimulation (tDCS). TMS delivers a larger current and is thought to cause neurons to fire (Ridding and Rothwell, 2007) while tDCS delivers a much smaller current and is believed to change the membrane potential of neurons (Nitsche et al., 2008; Priori et al., 2009) Both techniques have been used to enhance performance on tasks involved in a variety of cognitive processes in healthy and impaired adults (Miniussi et al., 2008; Nitsche et al., 2008; Williams et al., 2009; Nitsche and Paulus, 2011; Krause and Cohen Kadosh, 2013). While the evidence for brain stimulation improving function in healthy adults has been somewhat controversial (see Horvath et al., 2015 but also Price et al., 2015), its use as a treatment in patient populations with brain injury has been promising (Fregni and Pascual-Leone, 2007; Miniussi et al., 2008; Wong and Tsang, 2013).

More recently, research using brain stimulation to treat learning disorders, such as dyslexia and dyscalculia, has been called for (Cohen Kadosh et al., 2013; Krause and Cohen Kadosh, 2013; Vicario and Nitsche, 2013). While those with learning disorders do not have frank insult to the brain, they are believed to have altered brain activation in key brain regions when compared to typical adults (Pugh et al., 2001; Price and Ansari, 2013; Norton et al., 2014; Kucian and von Aster, 2015). The case for using neuromodulation to treat learning disorders is thus conceptually straightforward; stimulation to modify the activity in a brain region shown to be integral to the cognitive process of interest and differentially activated in the disordered population should normalize activation in that region and therefore normalize performance. However, identifying the brain regions integral to the process is not trivial, particularly in the case of reading.

Neuroimaging research has identified several brain regions that show altered function in individuals with dyslexia that may serve as potential targets of brain stimulation. Three brain areas in particular have consistently shown altered functionality compared to typical readers—the inferior frontal gyrus (IFG), temporo-parietal areas, and occipito-temporal areas (Richlan et al., 2009, 2011). Meta-analysis of neuroimaging studies suggest the IFG may be hyperactive in adults with dyslexia as a compensatory region, while temporo-parietal and occipito-temporal areas may be hypoactive, reflecting impaired processing during reading. Brain stimulation could be used to either enhance potential compensatory regions in an attempt to strengthen these networks or enhance areas that are consistently underactive in people with dyslexia in an attempt to normalize their function.

So far, neuromodulation studies examining the tool's potential to improve reading ability have stimulated regions shown to be underactivated in poor readers, and all have had some success (Costanzo et al., 2012, 2013; Turkeltaub et al., 2012; Heth and Lavidor, 2015; Thomson et al., 2015). However, the exact nature of the reading improvements has been somewhat inconsistent across studies. Turkeltaub et al. (2012) first demonstrated the potential for tDCS to be used as a treatment for low-to-average readers by showing improved reading fluency after stimulation to the left superior temporal gyrus (STG) compared to sham stimulation. These findings were corroborated by Costanzo et al.'s TMS studies (Costanzo et al., 2012, 2013) that found TMS to the left STG increased real word reading speed and text reading accuracy in both dyslexic and average readers. However, a later tDCS study by Thomson et al. (2015) was inconsistent with the findings of Turkeltaub et al. (2012). Thomson et al. (2015) stimulated an overlapping, but slightly superior region to that stimulated in Turkeltaub et al. (2012) in average readers and found that right hemisphere stimulation led to improvements on real word reading ability, not left hemisphere.

The Costanzo et al.'s TMS studies (Costanzo et al., 2012, 2013) showed that the particular section of the temporo-parietal region that is stimulated leads to specific results. In contrast to the increases in real word reading following STG stimulation mentioned above, stimulation to the more superior temporo-parietal cortex, specifically, the left inferior parietal lobe (L IPL), led to increases in pseudoword reading. While the lack of improvements in real word reading, the ultimate goal of reading therapy, is discouraging, stimulation to the IPL and surrounding areas merits further investigation. In particular, tDCS to the more superior aspects of the temporo-parietal cortex may lead to greater gains in reading ability compared to the precisely targeted stimulation of TMS given the diffusivity of tDCS. The superior portion of the temporo-parietal cortex including not just IPL, but also the angular gyrus (AG) and supramarginal gyrus (SMG), have been specifically related to smaller-grained grapheme-to-phoneme mapping (Pugh et al., 2000; Simos et al., 2001; Booth et al., 2003; Jobard et al., 2003; Cao et al., 2006; Bitan et al., 2007b; He et al., 2013), which developmental and cross-linguistic studies of reading suggest is important for the initial development of the reading network (Pugh et al., 2000; Cao et al., 2006, 2015; Richlan et al., 2011; Martin et al., 2015). Therefore, we propose that facilitation of small-grain grapheme-to-phoneme processing via modulation of activation in superior portions of the temporo-parietal cortex may lead to gains in multiple aspects of reading ability including reading fluency and improved grapheme-phoneme mappings in low-to-average ability adults.

This hypothesis is supported by second language learning studies with adults (Hashimoto and Sakai, 2004; Mei et al., 2014) and reading remediation studies with children (Temple et al., 2003; Simos et al., 2007; Meyler et al., 2008; Rezaie et al., 2011b) and adults (Eden et al., 2004) that show greater ability related gains in superior vs. inferior temporo-parietal cortex. Further, functional connectivity between the IPL in particular and regions involved in orthographic processing such as the fusiform gyrus (FG) has been demonstrated to be related to word reading ability (Koyama et al., 2011; Simon et al., 2013). Developmental studies have suggested that the strength of the connection between grapheme-to-phoneme processing regions such as the IPL and the orthographic processing regions such as the FG may be critical for the specialization of these orthographic processing regions, in line with interactive specialization models of development that have been extended to reading (Johnson, 2001; Schlaggar and McCandliss, 2007; Price and Devlin, 2011). Indeed, dyslexic readers tend to have reduced connectivity between these two regions compared to controls. In contrast, there is little evidence suggesting the connectivity between the STG and FG is crucial for reading ability (Horwitz et al., 1998; Pugh et al., 2000; Booth et al., 2008; Cao et al., 2008; Quaglino et al., 2008; van der Mark et al., 2011; Finn et al., 2014).

In order to test the hypothesis that tDCS to superior portions of the temporo-parietal cortex will lead to reading improvement for low ability readers, we stimulated the left IPL in low-to-average readers and measured their improvement on two reading tasks; single word reading efficiency and a rhyme judgment task. Both require the use of phonological and orthographic information, but in different ways. Single word reading efficiency requires articulating the phonological output from orthographic input. This skill has been shown to be related to both overall word reading ability and the ability to decode words based on grapheme-to-phoneme mappings (Adlof et al., 2006; Vellutino et al., 2007; Barth et al., 2009). Despite the orthographic processing component, neuroimaging studies have suggested that this skill is most related to parietal areas including the IPL and AG (He et al., 2013) typically implicated in grapheme-phoneme mappings, compared to areas involved in whole-word orthographic mappings. Further, training studies have shown that instruction in grapheme-to-phoneme mapping results in improvement in single word reading fluency (Ashmore et al., 2002; Simos et al., 2007) and these gains are related to activation in parietal areas (Rezaie et al., 2011a,b). The rhyme judgment task, in contrast, does not require articulation. Rather it requires the activation and memory of phonological representations, sometimes in the face of conflicting orthographic information. For this reason, this task is a measure of phonological working memory and the strength of the grapheme-to-phoneme maps needed to be activated to complete the task accurately. Behavioral studies have shown the rhyming task to be related to reading ability (Maclean et al., 1987; Ziegler and Goswami, 2005; Kovelman et al., 2012) and neuroimaging studies have shown activation in the left IPL to be related to ability on this task (Booth et al., 2003; Hoeft et al., 2006; Bitan et al., 2007a). Together, both tasks provide measures of orthographic and phonological processing that are related to activation in the left IPL. By using these two tasks, we can address how tDCS affects both the mental manipulation and articulation of phonological representation and therefore have a broader picture of what abilities can be impacted by tDCS.

## Methods

This study was carried out in accordance with the recommendations of the University of Texas at Austin Institutional Review Board with written informed consent from all subjects. All subjects gave written informed consent in accordance with the Declaration of Helsinki.

### Participants

In total, 100 right-handed 18–35 year-old native English speakers with normal or corrected-to-normal vision were screened for below average reading ability (< 100 standard score) as determined by the Sight Word Efficiency (SWE) subtest of the Test of Word Reading Efficiency (TOWRE; Torgesen et al., 1999) in line with Turkeltaub et al. (2012). All participants reported no history of neurological disorder, psychiatric disorder, significant head trauma, hearing loss, substance abuse, seizure or migraine, metal implants, and current pregnancy. Of the initial 100, 54 participants scored below average, however, 14 did not complete both days of the experiment, and were therefore not included in the sample. An additional four participants were excluded for scoring < 50% accuracy on behavioral measures. The remaining participants had at least average (>80 standard score) intelligence as measured by the Wechsler Abbreviated Scale of Intelligence (WASI; Wechsler, 2008). Scores on the SWE ranged from 74 to 99 pre-stimulation (within two standard deviations of 50th percentile performance of 100). Participants were randomly assigned to one of three groups, L IPL, right superior parietal lobe (R SPL), or Sham. Assignment to the R SPL group was done as part of an additional experiment not reported here. For the current experiment, the R SPL group served as a stimulation control condition in which participants received stimulation to a non-target region which complemented the no stimulation control condition fulfilled by the Sham group.

Of those who met all performance criteria, 11 (7 female) received real stimulation to the L IPL, 14 (6 female) received sham stimulation, and 11 (9 female) received real stimulation to the R SPL. Due to an imbalance in the run orders in the sham group, four participants were randomly eliminated for a final sample of 10 (4 female). One-way ANOVAs revealed no significant effects of group on all group characteristics and baseline measures as reported in Table 1.

**Table 1:** **Participant demographics and mean (SD) for performance for each participant group**.

	**L IPL**	**R SPL**	**Sham**
Age (years)	26.8 (5.5)	25.2 (3.5)	26.2 (4.9)
Gender (f)	7	8	4
IQ	112.1 (10.9)	111.0 (11.70)	109.3 (9.9)
Pre-single word reading	88.5 (8.4)	88.1 (7.6)	89.2 (7.9)
Pre-rhyme judgment RT (ms)	854 (124)	972 (147)	998 (193)
Pre-rhyme judgment accuracy (%)	91.6 (7.0)	88.4 (9.5)	87.9 (12.0)

*IQ measured by Wechsler Abbreviated Scale of Intelligence. Single Word Reading was measured by the Sight Word Efficiency subscale of the Test of Word Reading Efficiency. IQ and Single Word Reading have μ = 100, σ = 15*.

### Procedure

Participants took part in a single-blind, sham and stimulation controlled study comparing pre- and post-stimulation performance on two measures of reading ability: single word reading efficiency and rhyme judgment. Participants completed two sessions that took place 3–5 days apart. During the first session, participants completed standardized tests and baseline assessments of reading ability. During the second session, participants received either sham or real stimulation for 20 min, after which they completed an alternate form of the reading ability measures using different sets of stimuli. Alternate forms of the tasks were counterbalanced across participants.

### Transcranial direct current stimulation

Direct current was administered using a battery-driven DC stimulator device (NeuroConn) via two saline-soaked electrodes (5 × 5 cm; 25 cm^2^). The anode electrode was placed over either the L IPL (P3) or R SPL (CP4) according to the international 10–20 system for electroencephalography (EEG) electrode placement (Herwig et al., 2003). The cathode (return) electrode was placed over the contralateral supraorbital frontal region. This montage allows the source of effects of stimulation to be more reliably attributed to the anodal stimulation of the target site instead of the cathodal stimulation of the reference site, as suggested by Turkeltaub et al. (2012). During real stimulation, 1.5 mA of current (current density 0.06 mA/cm2) was delivered for 20 min. During sham stimulation, the machine ramped up to 1.5 mA for 30 s, then extinguished over a 5 s fade-out. Using this procedure allows participants to feel the initial sensations (e.g., tingling or itching) associated with stimulation without any after-effects of stimulation being induced (Nitsche and Paulus, 2000). These stimulation parameters replicate the parameters used in Turkeltaub et al. (2012) and are within the safety limits established in prior studies on humans and animals (Iyer et al., 2005; Nitsche et al., 2008; Bikson et al., 2009).

### Experimental tasks

#### Single word reading efficiency

Word reading efficiency was measured via the SWE subtest of the TOWRE. This test is a measure of ability to read real words accurately and quickly. Participants were given 45 s to read aloud as many of 104 words as possible. A standard score is determined by the number of words read correctly within 45 s, and this score was used as the metric of single word reading efficiency.

#### Rhyme judgment task

The ability to map orthography to phonology and phonological working memory were assessed with a rhyme judgment task in which participants were presented with a series of visual word pairs and asked to indicate whether the words rhymed or not. Word pairs were designed to manipulate orthographic and phonological similarity to ensure participants could not rely on orthography alone and phonological representations had to be used to accurately complete the task. There were two congruent conditions in which word pairs had either similar orthography and phonology (e.g., CAGE-RAGE) or not (e.g., TRIAL-FALL), and two incongruent conditions in which word pairs had either similar orthography but dissimilar phonology (e.g., PINT-MINT) or dissimilar orthography but similar phonology (e.g., GRADE-PAID) pairs. Each condition had 12 trials for a total of 48 trials in each session.

All words were monosyllabic, having neither homophones nor homographs and were matched across condition for written word frequency in children (Zeno, 1995) and the sum of written bigram frequency (Balota et al., 2007). Stimuli used in each version of the task were matched on average stimuli length, frequency, number of orthographic neighbors, and number of phonological neighbors (Balota et al., 2007).

Participants were asked to respond as quickly and as accurately as possible. The first word was presented for 800 ms followed by a 200 ms inter-stimulus interval and the presentation of the second word. Participants could respond as soon as the second word was presented up to 2500 ms after the onset of the word. After the participant responded, a red fixation cross appeared signaling the inter-trial interval. The task was self-paced and participants were able to control when the next trial began. Average reaction times (RT) to correct trials trimmed to include only responses within 2.5 standard deviations from an individual's average reaction time were used as the metric of rhyme judgment ability due to ceiling effects on accuracy.

### Analysis

Performance on each experimental task was submitted to a 3 (Stimulation group; L IPL, Sham, R SPL) × 2 (Time; Session 1, Session 2) mixed-model ANOVA in order to determine whether a measure showed a Group × Time interaction. Planned follow-up tests were conducted using separate 2 (Stimulation group; L IPL, Sham or R SPL) × 2 (Time; Session 1, Session 2) mixed-model ANOVAs to examine potential Group × Time interactions for the L IPL group compared to the two control groups (Sham, R SPL) separately.

## Results

### Single word reading

**Figure 1:**
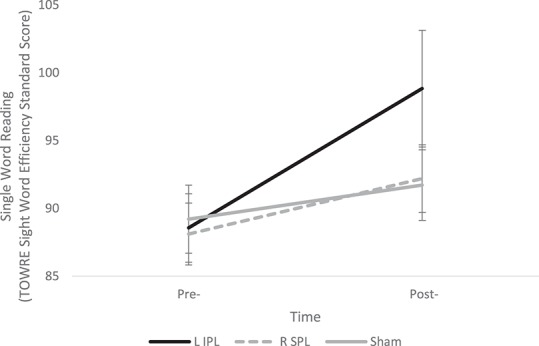
**Change in Single Word Reading Efficiency pre- and post-stimulation for each group**. A significant Group × Time interaction indicates the L IPL group showed significantly greater improvement than the R SPL and Sham groups. Error bars indicate one standard deviation.

The 3 × 2 ANOVA revealed a significant main effect of Time [*F*_(1, 29)_ = 24.68, *p* < 0.001] and significant Group × Time interaction [*F*_(2, 29)_ = 4.41, *p* = 0.021], indicating that although all groups showed changes in their performance over time, the groups differed in the magnitude of these changes. Follow-up tests revealed the L IPL group showed significantly greater improvement compared to the Sham group [*F*_(1, 19)_ = 7, *p* = 0.016] and a trend toward greater improvement compared to the R SPL group [*F*_(1, 20)_ = 4.22, *p* = 0.053]. One sample *t*-tests indicated that the L IPL and R SPL groups' improvement was significantly greater than 0 [L IPL: *t*_(10)_ = 4.17, *p* < 0.005; R SPL: *t*_(10)_ = 2.37, *p* < 0.05], while the Sham group did not show improvement [*t*_(9)_ = 1.72, *p* > 0.1] see Table 2 and Figure 1. The results of the current study result in an effect size (Cohen's d) of 1.57 for L IPL stimulation, greater than the 0.46 for left STG stimulation found by Turkeltaub et al. (2012).

**Table 2:** **Mean (SD) on reading measures pre- and post-stimulation**.

	**L IPL**		**R SPL**		**Sham**	
	**Pre**	**Post**	**Pre**	**Post**	**Pre**	**Post**
Single word reading	88.5 (8.4)	98.8 (14.3)	88.1 (7.6)	92.2 (8.3)	89.2 (7.9)	91.7 (8.2)
Rhyme judgment RT (ms)	854 (124)	811 (142)	972 (147)	885 (141)	998 (193)	820 (99)
Rhyme judgment accuracy (%)	91.7 (7.0)	94.7 (7.5)	88.4 (9.5)	89.6 (10.6)	87.9 (12.1)	90.0 (9.0)

*Single Word Reading has μ = 100, σ = 15*.

### Rhyme judgment

All participants performed well on the rhyme judgment task as indicated by high accuracy at Time 1 and Time 2. A 3 × 3 ANOVA did not reveal a main effect of Time or any Group × Time interactions (*p* > 0.1). One sample *t*-tests for each group individually showed that no group's gain in accuracy was significantly greater than 0 (*p* > 0.1). These findings indicate that there was neither a practice effect nor an effect of stimulation on accuracy, possibly due to ceiling effects.

**Figure 2:**
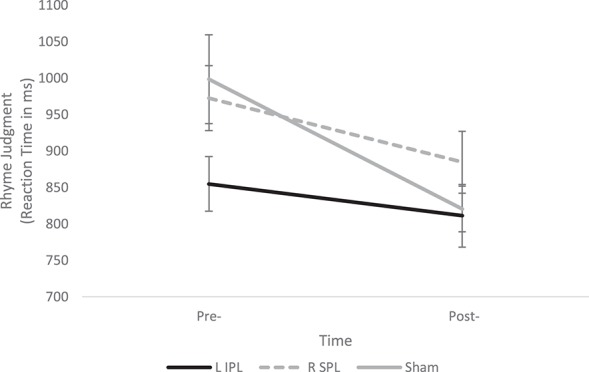
**Change in Rhyme Judgment performance pre- and post-stimulation for each group**. A significant Group × Time interaction indicates the Sham group showed significantly greater improvement than the L IPL and R SPL groups. Error bars indicate one standard deviation.

The 3 × 2 ANOVA again revealed a significant main effect of Time [*F*_(1, 29)_ = 28.9, *p* < 0.001] and significant Group × Time interaction [*F*_(2, 29)_ = 4.13, *p* = 0.026], indicating a change over time, but a group difference in the magnitude of the change. Follow-up tests showed the Sham group experienced significantly greater improvements in RT compared to the L IPL group [*F*_(1, 19)_ = 7.27, *p* = 0.014]. However, the R SPL group was not significantly different from either the Sham group [*F*_(1, 19)_ = 2.62, *p* > 0.1] or the L IPL group [*F*_(1, 20)_ = 1.69, *p* > 0.1]. *Post-hoc* one-sample *t*-tests, though, indicate that both the Sham and R SPL groups' improvement was significantly greater than 0 while the L IPL group did not improve [Sham: *t*_(9)_ = 2.54, *p* = 0.006; R SPL: *t*_(10)_ = 2.31, *p* = 0.044; L IPL: *t*_(10)_ = 1.74, *p* > 0.1] see Table 2 and Figure 2.

## Discussion

The goal of the current study was to assess whether stimulation of the L IPL improves multiple aspect of reading for low-to-average readers by measuring its impact on two tasks that tap into different subskills of reading. While L IPL stimulation did result in gains in single word reading efficiency, it resulted in relative impairment on the rhyme judgment task, demonstrating for the first time a significant positive and negative effect on two different tasks with the same stimulation parameters within the same group of participants. Although our results indicate stimulation to the L IPL may be a good site for improving reading fluency for low-to-average readers, the lack of improvement on the rhyme judgment task warrants caution in advocating the left IPL as a site to improve several aspects of reading.

The positive influence of L IPL stimulation on reading fluency measures for low-to-average readers was consistent with our hypothesis. Although there was a main effect of time, indicating there was a general practice effect for all groups, the L IPL stimulation resulted in greater improvement than the Sham or R SPL stimulation. Our finding that stimulation to the IPL led to greater improvements for low-to-average readers than previous reports of stimulation to the STG (Turkeltaub et al., 2012) are in line with studies indicating that single word reading fluency abilities depend on grapheme-to-phoneme mapping skills supported by the superior aspects of the temporo-parietal cortex (Ashmore et al., 2002; Simos et al., 2007; Rezaie et al., 2011a,b; He et al., 2013). This finding suggests that improvements in single word reading can result from increased grapheme-to-phoneme mapping abilities, even in adults. However, it should be noted that montage differences between the current study and that by Turkeltaub et al. (2012) could also account for the difference in effect size between the two studies. Turkeltaub et al. (2012) used a bilateral montage, meaning that the cathode or reference electrode was placed on the contralateral hemisphere (i.e., right STG). The results of that study cannot be attributed solely to facilitation of the left hemisphere, and the effects could have been due to alteration in the balance between the two hemispheres or even to inhibition of the right hemisphere. In contrast, in the current study, the cathode electrode was placed on the contralateral forehead. While we cannot rule out that the effects in the current study were due to inhibition of the frontal lobe, it is more reasonable to conclude that the effects are due to the modulation of activation in the stimulation site and surrounding areas. As Turkeltaub et al. (2012) themselves point out, it could be that unilateral stimulation may be more beneficial than bilateral stimulation. Further research should explore how montage affects the behavioral consequences of stimulation.

Our results that left hemisphere stimulation leads to improvement in SWE is in contrast to Thomson et al. (2015) who found that right, but not left, hemisphere stimulation led to behavioral improvements. These conflicting results are likely due to the difference in populations used in each study. In keeping with the Turkeltaub et al. (2012) findings that stimulation only led to improvements in low-to-average readers, the current study only used readers who had below average performance on the TOWRE. However, Thomson et al. (2015) used participants with a wide range of reading abilities. Because individual differences in skill have been shown to have an effect on the behavioral changes induced by tDCS, including individuals with a large range of reading ability may have diluted the effects for the left hemisphere stimulation in the Thomson et al. (2015) study. Future research should examine how individual differences in reading ability affect the impact of tDCS on behavioral performance.

In contrast to the expected results of tDCS on the SWE, the negative effect of L IPL stimulation on improvement on the rhyming judgment task in low-to-average readers was unexpected. Previous neuroimaging work with both children and adults has shown that increases in activation in the left IPL are associated with better performance on the rhyming task (Hoeft et al., 2006; Bitan et al., 2007a; Cao et al., 2015). If anodal stimulation to the L IPL did increase activation in that area as hypothesized, the stimulation group should have shown increases in performance following stimulation. However, research with anodal stimulation to the IPL in the context of working memory has shown that stimulation actually impairs performance relative to sham, particularly in low performers (Jones and Berryhill, 2012; Sandrini et al., 2012). Specifically, Sandrini et al. (2012) showed that anodal stimulation abolished practice effects on a working memory task. These findings are in line with the results of the current study; while the L IPL stimulation group did not perform worse after stimulation, they did not demonstrate practice effects as seen in the sham group. The rhyming task involves phonological working memory when the phonological representation of the first word must be held in mind until the second word is presented and the two phonological representations can be compared to make a rhyme decision. Therefore, the effects of stimulation on working memory abilities may have prevented improvements on the task. Previous work examining the effect of stimulation to the parietal lobes has suggested that stimulation interferes with working memory by creating an imbalance of activation between the two hemispheres and interfering with the natural inter-hemispheric inhibition that occurs in the absence of stimulation (Sparing et al., 2009; Sandrini et al., 2012; Park and Friston, 2013; Krause and Cohen Kadosh, 2014). Inter-hemispheric inhibition may explain the seemingly contradictory results of the current study. As reading skills develop, activation during reading becomes more lateralized to the left hemisphere with the right hemisphere playing a decreasing role in reading (Turkeltaub et al., 2003; Eden et al., 2004; Shaywitz et al., 2004). Simulation causing disruptions to inter-hemispheric inhibition may thus be less likely to affect single word reading abilities, and therefore, we can expect improvements on single word reading tasks such as those seen in the current study. However, previous research has shown that low-to-average and dyslexic readers tend to have more bilateral activation during reading tasks with a phonological working memory component (Milne et al., 2002; Illingworth and Bishop, 2009; Xu et al., 2015). Given that the subjects in the current study were all low-to-average readers, it could be the case that the disruption to inter-hemispheric inhibition in the parietal lobes was particularly detrimental to their phonological working memory abilities, preventing the expected practice effects on the rhyming task. Our findings highlight the importance of considering the impact that individual differences may have on neural processing and subsequently the effects of tDCS.

From a clinical perspective, perhaps the most important finding from the current study is that tDCS can positively impact one skill while negatively impacting another. Our results underscore the importance of including multiple tasks that potentially tap into different underlying cognitive processes in order to assess whether the potential costs of stimulation outweigh the potential gains. Assessing multiple tasks becomes especially important when considering whether tDCS should be recommended as a treatment. In the current study, the cost in practice effect on speed during a rhyme judgment task is probably worth the gains seen in single word reading for low-to-average readers; accuracy was not affected and speed did not decrease after stimulation. However, this population was low-to-average in skill, not impaired. The cost to benefit ratio may increase as reading skill decreases. Further studies examining how individual differences impact the effects of tDCS on multiple reading tasks are needed before being able to advocate for tDCS as a treatment for reading disabilities.

Further, our findings that tDCS had a differential effect on two aspects of reading in low-to-average readers has implications for the design of future tDCS studies. In the current study, both tasks were reading-related, but the differences between tasks in the working memory component are in line with the literature that anodal tDCS does not have the expected positive effect on working memory abilities. These results support the idea that tDCS can affect cognitive processes differently, depending on how the target or surrounding brain area is involved in a given cognitive process. Future research is still needed to determine the circumstances in which the conventional idea that anodal stimulation leads to enhancement of activation and behavior while cathodal stimulation leads to inhibition of activation and behavior holds true (De Berker et al., 2013; Bestmann et al., 2015). When selecting target sites, the areas' involvement in multiple cognitive processes should be considered. The impact of stimulation on each of these processes should then be examined so that we might better understand whether tDCS can influence cognitive processes differently.

### Limitations

The current study used a between subjects design, meaning that the different stimulation groups were composed of different individuals. While the groups were equated on task behavioral abilities, it is virtually impossible to equate them on all factors that could potentially impact the effects of stimulation. For example, other research groups have shown that individual differences in physiological measures such as skull thickness, and levels of certain hormones and neurotransmitters such as GABA, can affect the way stimulation affects an individual (Krause and Cohen Kadosh, 2014). Additionally, measures of non-reading related neurocognitive abilities were not collected. It is possible that differences in other cognitive processes between the two groups may have affected results. While the current study did not control for such measures, the use of random assignment to group and ensuring the group was matched on task performance should minimize potential confounds from these factors.

Finally, as with all tDCS studies, without the use of neuroimaging techniques such as structural or functional magnetic resonance imaging (fMRI), we are unable to confirm that the stimulated area was in fact the targeted area. Individual differences in anatomy may have led to differences in how well the stimulation site aligned with the brain regions that are actually used to perform the tasks. Similarly, due to the distributed effects of tDCS we cannot make strong conclusions about whether the results are due to stimulation to the targeted region or surrounding and connected regions without neuroimaging measures. Future research with tDCS would benefit from using neuroimaging methods to have more precisely located targets and a better understanding of the locations that were actually affected in order to develop the most effective treatment methods.

## Conclusions

Our study provides important cautionary evidence for the use of tDCS as a treatment for low reading ability. Although stimulation to the left IPL led to greater improvements in reading fluency than those previously demonstrated with a different stimulation site (2012), we also found a negative effect on another subcomponent of reading in low-to-average readers, i.e., rhyming two visually presented words. These positive and negative effects on two different subcomponents of reading were demonstrated using the same stimulation parameters within the same participants. These results stress the need for further research examining the effect of a set of stimulation parameters on complementary skills so that potential users of tDCS as a therapy can accurately weigh the costs and benefits of the treatment.

## Author contributions

MR and JB conceived and designed the experiments. MR and JY performed the experiments. JY and JB analyzed and interpreted the data. JY drafted the manuscript. JY, MR, and JB performed a critical review of the manuscript. All the authors read and approved the final version of the manuscript.

### Conflict of interest statement

The authors declare that the research was conducted in the absence of any commercial or financial relationships that could be construed as a potential conflict of interest.
